# Miliary Tuberculosis-Related Acute Respiratory Distress Syndrome Complicated with Hemophagocytic Lymphohistiocytosis Syndrome

**DOI:** 10.1155/2019/9501610

**Published:** 2019-05-30

**Authors:** Sz-Jiun Shiu, Ting-Ting Li, Bor-Jen Lee, Pin-Kuei Fu, Chen-Yu Wang, Sz-Iuan Shiu

**Affiliations:** ^1^Department of Pediatrics, Chung Shan Medical University Hospital, Taichung, Taiwan; ^2^Division of Cardiovascular Surgery, Department of Surgery, Kaohsiung Chang Gung Memorial Hospital, Kaohsiung, Taiwan; ^3^Department of Critical Care Medicine, Taichung Veterans General Hospital, Taichung, Taiwan; ^4^Division of Gastroenterology and Hepatology, Department of Internal Medicine, Taichung Veterans General Hospital, Taichung, Taiwan

## Abstract

Acute respiratory distress syndrome (ARDS) and hemophagocytic lymphohistiocytosis (HLH) are accompanied with poor outcome and high mortality when miliary tuberculosis is a causative pathogen for both of them. A patient complicated with ARDS and HLH is unusual in critical care, and few case reports are present in PudMed. Besides, the relationship between HLH and ARDS is still unknown and has not been reviewed in the literature. In this report, we present the case of a 74-year-old Taiwanese woman suffering from pulmonary tuberculosis and miliary tuberculosis, and she developed ARDS and HLH on the 3rd day after admission. We arranged serial laboratory examination, various serum markers, bone marrow aspiration, and bronchoscopy with alveolar lavage for survey; we prescribed empirical antibiotics and antituberculosis medication soon after alveolar lavage showing positive acid-fast stain. She was extubated on hospital day 31 and discharged on hospital day 73. In conclusion, early diagnosis and intervention for underlying disease and intensive bundle care for multiorgan failure are crucial for both ARDS and HLH.

## 1. Introduction

Both acute respiratory distress syndrome (ARDS) and hemophagocytic lymphohistiocytosis (HLH) are accompanied with poor outcome and high mortality. Miliary tuberculosis is a causative pathogen for both of them. There are very few literatures discussing about patients complicated with ARDS and HLH. Herein, we present a patient diagnosed with miliary tuberculosis-related ARDS combined with HLH and discuss about the relationship among miliary tuberculosis, ARDS, and HLH.

## 2. Case Presentation

A 74-year-old woman without relevant medical history presented with intermittent fever for more than one month. She also mentioned body weight loss about 18 kilograms and dry cough in recent half year. She did not travel in recent one year. She was admitted to local hospital and was transferred to our hospital for survey.

On physical examination at admission, her temperature was 37.3°C, heart rate 101 beats/min, respiratory rate 16 breaths/min, and blood pressure 142/54 mmHg. Pulmonary examination revealed accessory muscles use and bilaterally diffuse rales. Abdominal examination showed palpable spleen contour while palpation without muscles guarding or rebounding pain. The rest of the physical examination was unremarkable.

**Figure 1:**
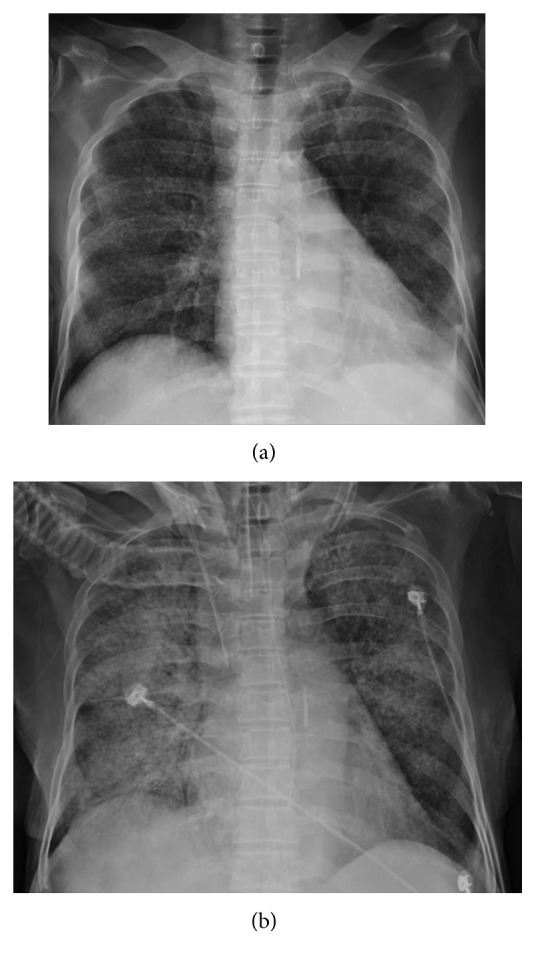
(a) CXR of the 1st day after admission. (b) CXR of the 3rd day after admission (the day of intubation).

**Figure 2:**
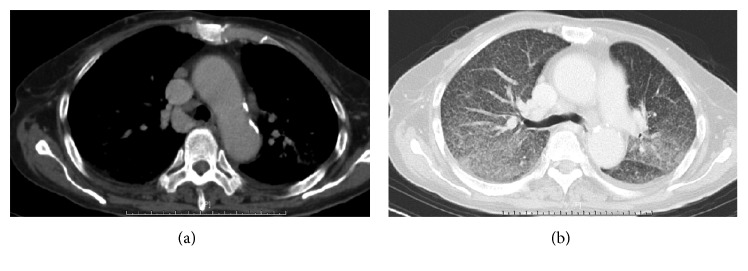
(a) Computed tomography (CT) of chest in soft tissue window revealed lymphadenopathy over mediastinum and (b) multiple tiny nodules along with alveolar infiltration in lung window over bilateral upper lung fields, diagnosed as military tuberculosis combined with pulmonary tuberculosis.

Laboratory examination revealed a total leukocyte count of 19,900 cells per *μ*L and a platelet count of 133,000 per *μ*L. Her alkaline phosphate was 481 U/L, serum total bilirubin 3.2 mg/dL, and lactate dehydrogenase 311 U/L. Her serum transaminases, internal normalized ratio (INR), and the activated partial thromboplastin time (aPTT) were all within normal range on initial presentation. Initial acid-fast stain of sputum, stool, and urine was negative. Chest X-ray showed multiple tiny nodules over bilateral lung field with alveolar infiltration over bilateral upper lobes, suspected as pulmonary tuberculosis with miliary tuberculosis (Figure 1(a)). Computed tomography from local hospital revealed multiple tiny nodules over bilateral lung fields, hepatosplenomegaly, and lymphadenopathy over omentum plus mediastinum, which indicated disseminated tuberculosis (Figures 2(a) and 2(b)).

On the 3rd day of hospitalization, progressive orthopnea developed with paradoxical respiration (Figure 1(b)). Oxyhemoglobin saturation by pulse oximetry (SpO_2_) was 83% in room air. Emergent intubation was performed, and she was transferred to the respiratory intensive care unit (RICU) for resuscitation. Serial laboratory examination revealed leukocytosis with left shifting, INR > 10 ratio, aPTT > 100 seconds, fibrinogen 137.2 mg/dL, ferritin 980 ng/mL, triglyceride 110 mg/dL, sIL-2 receptor > 5000 pg/mL, and PaO_2_/FiO_2_ of arterial blood gas 55.1. Chest X-ray revealed newly developed symmetrically alveolar infiltration over bilateral lung field. Severe septic shock with acute respiratory distress syndrome was diagnosed. HLH was suspected due to splenomegaly, decreased fibrinogen, elevated ferritin, and sIL-2 receptor. Bone marrow biopsy was needed for definite diagnosis but held because her condition was unstable, and her family also hesitated due to high risk. Early goal-directed therapy was initialized with empirical antibiotics of Tazocin. Besides, ventilator setting was lung protective strategy. In addition, serum markers were negative for HIV-1, HIV-2, hepatitis B surface antigen, hepatitis C virus IgG antibody, influenza virus type A antigen, influenza virus type B antigen, IgG and IgM antibodies to cytomegalovirus, and herpes simplex virus. The Epstein-Barr virus (EBV) capsid antigen IgG antibodies were positive while EBV early antigen and nuclear antigen IgG antibodies and EBV IgM antibodies were all negative, indicating prior infection. We did not check EBV DNA from the patient's serum by polymerase chain reaction (PCR). On the 4th day, we arranged bronchoscopy for bronchoalveolar lavage fluid, and the result showed positive acid-fast stain 4+ and no bacteria were found. PCR for DNA of tuberculosis was also positive. We shifted antibiotics to antituberculosis medication of HERZ (isoniazid, ethambutol, rifampin, and pyrazinamide) plus levofloxacin immediately. On the 12th day, definite culture showed *Mycobacterium tuberculosis* which was sensitive to all antituberculosis medication. Bone marrow aspiration on this day confirmed diagnosis of HLH, and blood smear showed phagocytosis of macrophages without granular formation and negative acid-fast stain. We diagnosed HLH by fulfilling 5 out of 8 criteria of HLH in 2004. On the 31st day, she was extubated and finally she was discharged from our hospital after following for 73 days.

## 3. Discussion

HLH could be classified into primary and secondary forms. An underlying genetic mutation is found in only 40% of all primary HLH patients [1]. The secondary form of HLH is also called macrophage activation syndrome, which could be associated with chronic inflammatory diseases, especially rheumatic disorders, and inflammatory bowel diseases [2]. Secondary HLH could affect people in all ages and be triggered by many factors, including infection, malignancy, or autoimmune disorders. The median survival of familial HLH was usually less than two months without treatment [3] compared to the mortality rate of secondary HLH exceeding 50% [4]. The etiology regarding to infection-associated HLH included virus, bacteria, tuberculosis, parasite, and fungus. The most common pathogen was EBV [5]. Priscilla et al. reviewed 36 cases of tuberculosis associated with HLH. 83% of patients had evidence of extrapulmonary tuberculosis while nearly 42% of patients had disseminated tuberculosis at presentation. The mortality was nearly 50% in this review [6].

In JAMA of 2012, the Berlin definition of ARDS was published by panel of experts conducting a meta-analysis [7]. In the meta-analysis, the stages such as mild, moderate, and severe ARDS were associated with increased mortality (27%, 32%, and 45%, respectively) and increased median duration of mechanical ventilation in survivors with significance.

There were only few case reports describing patients co-existed with ARDS and HLH at the same time in literatures. An author illustrated patients with ARDS and HLH that resulted from infection by scrub typhus [8]. Another author presented a patient with SLE who suddenly died of associated ARDS with accidental findings suggestive of hemophagocytic syndrome documented by autopsy [9]. The exactly pathophysiologic mechanism for ARDS and HLH is not fully understood nowadays, and the hypothesis of HLH nowadays includes three major components [10–12], as follows:

1. The defect of perforin-mediated cytotoxic T lymphocytes (CTLs) and/or nature killer (NK) cells are the pathological causes, which might be due to known genetic factors and/or undiscovered factors. The preexisting genetic defects of CTLs and/or NK cells might be stimulated by multiple trigger factors, including infection, malignancy, or autoimmune disorders.
2. The hyperstimulated cytokines, including INF-*γ*, IL-12, and IL-18, could magnify the cascade of cell proliferation, including lymphocytes and macrophages. The CTLs and/or NK cells loss the ability to eliminate the circulating antigens via the perforin-mediated cytotoxicity, and continuous antigen-mediated stimuli results in hyperproliferation of CTLs and/or NK cells along with hypersecretion of unregulated and stimulated cytokines into blood stream.
3. The hyperproliferated CTLs and macrophages combined with hypercytokinemia result in clinical syndromes of HLH. Hyperproliferated CTLs and macrophages infiltrating into multiple organs and activated histiocytes residing in organs cause CNS symptoms, organomegaly, cytopenia, and hemophagocytosis in bone marrow, spleen, and lymph nodes. Hypersecreted cytokines, including IL-1, IL-6, and TNF-*α*, cause systemic inflammatory responses.

The pathogenesis of ARDS to our understanding involved altered permeability of alveolar endothelial and epithelial barrier, uncontrolled inflammation, overexcited leukocytes, and cytokines, including TNF-*α*, IL-1, thrombin, and microbe-related toxins [13]. While patients with ARDS were intubated and maintained by the ventilator, the ventilator-associated lung injury also aggravated ARDS. The mechanism included increased cytokine of IL-1*β*, IL-6, *β*-catenin, and TNF-*α*, increased concentration of IL-8 and TGF-*β*, and recruitment of neutrophiles and pulmonary alveolar macrophages [14].

The majority of the participants in HLH were CTLs, NK cells, and macrophages, which was different from ARDS, including neutrophiles and resident macrophages in pulmonary alveolus. In addition, the cytokines causing cascaded inflammatory responses in HLH were mainly INF-*γ*, IL-12, IL-18, IL-1, IL-6, and TNF-*α* while the cytokines in ARDS were IL-1*β*, IL-6, IL-8, *β*-catenin, TNF-*α*, and TGF-*β*. Neither pathogenomic cells nor secreted cytokines can account for the relationship between ARDS and HLH. It might need further studies to investigate whether ARDS and HLH were related to each other or they were independent in relationship. In our patient, her condition progressed with ARDS and HLH nearly at the same time on the 3rd day after admission. Therefore, we concluded that our case is a miliary tuberculosis patient complicated with ARDS and HLH at the same hospital course, respectively. Delayed diagnosis might be the possible reason for our patient to develop both ARDS and HLH. If HLH was infection related, the priority of therapy should be antibiotics first rather than chemotherapy. Fortunately, our patient was responsive to antituberculosis medication, and there was no need for chemotherapy. However, a diagnosis of primary HLH should always be excluded, and with improved molecular diagnostics, it is recognized that cases of adult-onset HLH that had previously been considered secondary HLH may represent primary HLH with an underlying mutation in the PFR1 gene [15, 16]. In conclusion, to identify infectious pathogens, underlying autoimmune disease or malignancy earlier, and prompt treatment for underlying disease would be the key to the survival of patients.
